# Understanding access to novel high-cost cancer therapies across Canada: a national survey of pediatric oncology providers

**DOI:** 10.3389/fped.2026.1793250

**Published:** 2026-05-20

**Authors:** Kriti Kumar, Avram Denburg, Celine Cressman, Derek S. Tsang, Marcel Romanick, Myanca Rodrigues, Paul J. Gibson

**Affiliations:** 1 Division of Haematology/Oncology, Department of Paediatrics, The Hospital for Sick Children, Toronto, ON, Canada; 2 Child Health Evaluative Sciences, SickKids Research Institute, Toronto, ON, Canada; 3 Radiation Medicine Program, Princess Margaret Cancer Centre, University Health Network, Toronto, ON, Canada; 4 Provincial Pharmacy Services, Alberta Health Services, Edmonton, AB, Canada; 5 Department of Health Research Methods, Evidence, and Impact, McMaster University, Hamilton, ON, Canada; 6 Biostatistics Unit, Father Sean O’Sullivan Research Centre, St Joseph’s Healthcare Hamilton, Hamilton, ON, Canada; 7 Division of Hematology/Oncology, Department of Pediatrics, McMaster Children’s Hospital, Hamilton, ON, Canada

**Keywords:** access, disparities, health systems, novel anti-cancer agents, pediatric oncology

## Abstract

**Introduction:**

Many evidence-informed high-cost therapies for childhood cancer, including targeted drugs, proton beam therapy (PBT), and cellular therapy (CT) are not publicly funded in Canada. This study aimed to identify disparities and barriers to access, and key health policy changes to improve access to high-cost therapies for children.

**Methods:**

We conducted an online cross-sectional vignette-based survey among pediatric oncology providers at 16 Canadian pediatric oncology centres. Vignettes explored access to evidence-informed but not universally funded therapies: blinatumomab for low-risk relapse of B-cell acute lymphoblastic leukemia (B-ALL), larotrectinib for TRK-fused soft tissue sarcoma, PBT for unresectable head-and-neck sarcoma, and tisagenlecleucel for first relapse of B-ALL in a patient with Down syndrome. The primary outcome was access to each therapy, defined as the patient's ability to receive the specified therapy; secondary outcomes included time to access, funding sources, and perceived barriers.

**Results:**

Seventy participants were enrolled from 15 centres, with 68 (response rate = 97%) completing at least one survey question. Most respondents were pediatric medical oncologists (31/68, 45.6%). Provider-reported access rates were 89% for blinatumomab (*n* = 35), 79% for larotrectinib (*n* = 27), 59.2% for PBT (*n* = 30), and 94% for tisagenlecleucel (*n* = 30). Key barriers to accessing blinatumomab, PBT, and tisagenlecleucel included patient/family inability to travel, and the economic and psychosocial impact of travel. High cost of larotrectinib was a notable barrier.

**Conclusions:**

Access to evidence-informed cancer therapies for Canadian children remains variable. Universal funding, simplified approval processes, and the establishment of Canadian PBT centres to reduce travel burden, would ensure timely, equitable access to high-cost therapies.

## Introduction

In Canada's publicly funded, provincially administered healthcare system, access to novel cancer-directed therapies is often inequitable. Innovative therapies, including targeted drugs, proton beam therapy (PBT), and cellular therapy (CT), have continued to expand their indications in pediatric oncology. Despite compelling evidence for such novel therapies, many are high-cost and not currently publicly funded. In Canada, clinical data regarding novel therapies are first submitted to Health Canada for regulatory approval for sale and marketing. Once commercially available in Canada, products may be used for off-label indications. Concurrently, a submission can be made to the Canada Drug Agency (CDA), which reviews the therapy and makes recommendations regarding reimbursement to federal, provincial, and territorial health care funders (except Quebec). Provinces and territories may differ in their implementation of CDA recommendations, which can result in discrepant reimbursement decisions for novel therapies across jurisdictions.

Cancer therapies, including targeted agents and immunotherapy, are advancing in paediatric oncology. Despite growing evidence for their effectiveness, major barriers remain in delivering these treatments to patients, emphasizing the need for effective implementation strategies. In Canada, data on children's access to innovative cancer therapies is limited, though global disparities have been documented (1). It has been shown that for underserved populations in the US, therapies that are both effective and indicated, such as blinatumomab and CT, were not offered due to prohibitive consumer costs and logistical complications (2). Additionally, higher median household income, educational attainment, and private insurance have been associated with increased PBT use (3–5). In Canada, there are no proton beam facilities and CT centres are limited to five large urban centres across the country, creating further complexity by necessitating extended travel (6–8). Patients may experience financial toxicity from travel for PBT, with provinces providing inconsistent financial support (7). Exposure to poverty and lower socioeconomic status have also been shown to impact selection of certain therapies and ultimately relapse rates (2–5, 9).

To understand disparities in access to these high-cost innovative therapies, we devised an online survey to explore access to the following evidence-informed, but not universally funded therapies:

1. blinatumomab for patients with low risk first relapse of B-cell acute lymphoblastic leukemia (B-ALL) (10)
2. larotrectinib for pediatric TRK-fusion positive tumours (11)
3. PBT for head and neck sarcomas in children (8)
4. tisagenlecleucel for patients with Down syndrome and first relapse of B-ALL (12, 13)

We sought to characterize barriers in access to novel cancer therapies, with the aim of identifying targeted health policy and system changes that can improve patient access to current and future therapeutic innovations for childhood cancers.

## Materials and methods

### Study design and participants

We conducted an anonymous online survey among English-speaking pediatric oncology professionals at all 16 Canadian pediatric oncology centres. Eligible participants included pediatric medical oncologists, radiation oncologists, oncology pharmacists, drug access facilitators, hematopoietic stem cell transplant and CT physicians, and pediatric oncology nurse practitioners. No personal identifiers were collected.

The online survey was distributed from June to September of 2024 via Research Electronic Data Capture (REDCap). Pediatric oncology professionals at all 16 pediatric oncology centres in Canada were contacted directly by email and/or through email distribution lists of: the ACCESS (Advancing Childhood Cancer Experience, Science & Survivorship) network, C17 Council, and the Canadian Association of Radiation Oncologists (CARO). Monthly reminders were sent to those contacted directly. The study was approved by McMaster University's Health Sciences Research Ethics Board. The introductory text to the survey included that participation was voluntary, anonymous, and could be discontinued at any time without consequences. By proceeding with completion of the survey, participants indicated their implied consent.

### Instrument

The survey was developed in 2023 according to published guidelines by Burns et al., using a focus group of study investigators, expert opinion, and literature review (14). Each of the vignettes was piloted with at least two experts in the field, and the time to complete the survey was approximately 20 min. No compensation was provided to respondents for participation. The survey is included as a Supplementary File 1.

The survey consists of vignette-based stems, and the items generated in the survey were developed by consensus between the study investigators. The vignettes encompass inpatient therapy with blinatumomab for a child with low risk first relapse of B-ALL, outpatient oral therapy with larotrectinib for a child with TRK-fusion positive soft tissue sarcoma, PBT for an unresectable head-and-neck sarcoma, and tisagenlecleucel for a patient with Down's syndrome and first relapse of B-ALL. Vignettes were standardized for providers across provinces and territories. Selection of these therapies was based on relevance for treatment of children and youth with cancer, available evidence supporting the novel therapy, current Health Canada approved indications, and corollary potential for impact on pediatric patients in Canada (8, 10–13). These therapies also represent general classes of high-cost therapies being increasingly used in pediatric oncology, including immunotherapy (blinatumomab), targeted agents (larotrectinib), local control therapies (proton beam therapy), and cellular therapy (tisagenlecleucel). At the time of study design larotrectinib and PBT had received Health Canada approval for the indications in the vignettes (15, 16). The Health Canada approved indications for tisagenlecleucel only included pediatric and young adult patients up to and including 25 years of age with B-ALL who had refractory disease, had relapsed after allogeneic stem cell transplant (SCT) or were otherwise ineligible for SCT, or had experienced second or later relapse (17). For blinatumomab, the only Health Canada approved indication was the treatment of adults with Philadelphia chromosome-negative relapsed or refractory B-ALL (18).

Participants were invited to complete any portion of the survey relevant to their clinical practice and interests and were presented with the questions surrounding the vignette after selecting that the vignette was relevant to them. Participants could complete questions related to 0–4 vignettes depending on their clinical practice.

### Statistical analysis

All surveys, whether complete or partially completed, were included in the analysis. Analyses were descriptive. The primary outcome was provider-reported access to each innovative therapy explored in the vignettes, summarized overall, by province/territory. Access was defined as the ability of the patient to receive the specified therapy, at no cost to the patient/family for blinatumomab, tisagenlecleucel, and PBT, and minimal cost to the patient/family for larotrectinib. Secondary outcomes included perceived barriers to accessing novel therapies, indications for which novel therapies were obtained, sources of funding, and time to access. Time to access was defined as the time until the medication ready for administration for blinatumomab and larotrectinib, and until approval was obtained for PBT and tisagenlecleucel. Categorical variables (e.g., respondent characteristics, barriers, funding mechanisms) were summarized using frequencies and percentages. Continuous data (e.g., respondent characteristics, barriers, funding mechanisms) were summarized using means and standard deviations (SD). Comparisons across provinces/territories and centres were presented descriptively without formal hypothesis testing. As participants could complete between zero and four vignettes, descriptive statistics were performed per respondents in each vignette. Only available responses were analyzed (complete-case analysis). Imputation for missing data was not completed. All analyses were conducted using Stata SE version 17 (StataCorp, College Station, Texas) (19).

## Results

### Respondents

Seventy participants representing 15 out of 16 (94%) pediatric oncology centres provided consent to participate; 68/70 individuals (97%) completed at least a portion of the survey. Total respondents per vignette were 35 for blinatumomab, 27 for larotrectinib, 30 for proton therapy, and 30 for tisagenlecleucel. Participant demographics are summarized in Table 1.

**Table 1:** Participant demographics.

Variable	Number (%)
	*n* = 68
Primary Role	
Pediatric medical oncologist	31 (45.6%)
Radiation oncologist	7 (10.3%)
Oncology pharmacist	10 (14.7%)
Hematopoietic stem cell transplant/cellular therapy physician	3 (4.4%)
Nurse Practitioner/Advanced Practice Nurse	7 (10.3%)
Other*	3 (4.4%)
Region	
Western Canada**	14 (20.6%)
Ontario	29 (42.6%)
Quebec	14 (20.6%)
Atlantic Provinces***	4 (5.9%)
Institution	
British Columbia Children's Hospital	3 (4.4%)
Stollery Children's Hospital	4 (5.9%)
Alberta Children's Hospital	2 (2.9%)
Saskatoon Cancer Centre	5 (7.4%)
CancerCare Manitoba	1 (1.5%)
Children's Hospital, London Health Sciences Centre	1 (1.5%)
McMaster Children's Hospital	8 (11.8%)
Hospital for Sick Children	16 (23.5%)
Kingston General Hospital	1 (1.5%)
Children's Hospital of Eastern Ontario	2 (2.9%)
CHU de Sherbrooke	1 (1.5%)
CHU Sainte-Justine	9 (13.2%)
Montreal Children's Hospital	3 (4.4%)
IWK Health Centre	3 (4.4%)
Janeway Children's Health & Rehabilitation Centre	1 (1.5%)
Estimated Percentage of Patients Routinely Cared for Who Are Pediatric (Under 18 Years)	
20–39%	2 (2.9%)
40–59%	1 (1.5%)
60–79%	3 (4.4%)
80–100%	51 (75.0%)
Less than 20%	4 (5.9%)
Years in Practice	
>20 years	16 (23.5%)
11–15 years	5 (7.4%)
16–20 years	13 (19.1%)
6–10 years	13 (19.1%)
Up to 5 years	13 (19.1%)
Gender	
Female	16 (23.5%)
Male	38 (55.9%)
Non-binary	1 (1.5%)
Prefer not to answer	6 (8.8%)

* Other primary roles included research unit managers and pediatric neuro-oncologist/neurologist.

** Western Canada included British Columbia, Saskatchewan, Alberta, and Manitoba.

*** Atlantic Canda included Nova Scotia and Newfoundland and Labrador.

### Reported access to blinatumomab, larotrectinib, PBT, and CT

The percentage of time participants reported they could access blinatumomab, larotrectinib, PBT, and tisagenlecleucel for the indications in each vignette are summarized in Table 2, separated by province. Access was defined as the ability for a patient to receive the specified therapy. In addition to access for the specific indication outlined in the vignette, participants also reported additional indications that each therapy could be obtained for, summarized in Table 3.

**Table 2:** Reported access to each therapy overall and separated by province.

Province	BlinatumomabMean (SD)%*N* = 35	LarotrectinibMean (SD)%*N* = 27	Proton TherapyMean (SD)%*N* = 30	TisagenlecleucelMean (SD)%*N* = 30
Total	89.0 (23.3)	79.0 (29.5)	59.2 (30.0)	94 (11.7)
Alberta	87.3 (24.8)	99 (1.7)	82 (27.7)	96.7 (5.7)
British Columbia	80.3 (26.7)	76.7 (25.2)	63.5 (19.1)	70 (28.3)
Manitoba	100 (NA)	76 (NA)	100 (NA)	74 (NA)
Nova Scotia	75 (35.4)	58 (46.7)	33.3 (15.3)	100 (0)
Ontario	96.4 (8.7)	82.5 (23.6)	42.4 (22.2)	98.5 (3.4)
Quebec	70 (44.7)	64.7 (52.8)	68.5 (41)	95 (10)
Saskatchewan	100 (0)	100 (NA)	72.3 (25.4)	91.5 (12)

SD, standard deviation.

**Table 3:** Summary of indications that each therapy in the vignettes could be obtained for.

Indications	*N* (%)
Blinatumomab	N = 35
First relapse of B-ALL, MRD negative at End of Induction	32 (91.4%)
First relapse of B-ALL, MRD positive at End of Induction	32 (91.4%)
Infant KMT2A-rearranged B-ALL	24 (68.6%)
Bridging of front-line B-ALL therapy due to toxicity	29 (82.9%)
Salvage therapy for refractory B-ALL	31 (88.6%)
Larotrectinib	
*Disease Indication*	*N* = 27
Infantile fibrosarcoma	14 (51.9%)
Other TRK-fusion positive metastatic sarcomas	17 (63.0%)
TRK-fusion positive CNS tumors	15 (55.6%)
CNS tumors without a documented TRK-fusion	5 (18.5%)
Papillary thyroid cancer	6 (22.2%)
*Phase of Therapy for TRK-positive tumors*	*N* = 27
Metastatic disease	12 (44.4%)
Localized tumour	8 (29.6%)
Primary resection or radiation is not possible	11 (40.7%)
First-line monotherapy	11 (40.7%)
Monotherapy for TRK fusion positive tumours that have failed to have a response with all standard of care therapies	14 (51.9%)
Adjuvant use of larotrectinib with conventional chemotherapy as first-line therapy for TRK fusion positive tumours	9 (33.3%)
Adjuvant use of larotrectinib with conventional chemotherapy after failure of all standard of care therapies	9 (33.3%)
Proton Therapy	N = 30
Head-and-neck solid tumours	29 (96.7%)
CNS tumours	29 (96.7%)
Ocular tumours	27 (90.0%)
Patients with cancer predisposition syndromes (increased radiosensitivity)	17 (56.7%)
Retroperitoneal tumours	18 (60.0%)
Pelvic and genitourinary tumours	20 (66.7%)
Tisagenlecleucel	N = 30
Second or greater relapse of B-ALL following hematopoietic stem cell transplant	23 (76.7%)
First relapse of B-ALL in patients with Down's Syndrome	24 (80.0%)
CNS positive relapse of B-ALL in very young patients (<3 years)	14 (46.7%)
Primary refractory B-ALL	23 (76.7%)
B-ALL with positive MRD at the end of consolidation	13 (43.3%)

CNS, central nervous system; MRD, minimal residual disease; B-ALL, B-cell acute lymphoblastic leukemia.

### Time to access, processes, and transportation

Providers were more likely able to access blinatumomab within 2 weeks (32/35, 91%), while the majority of respondents reported time to access of 5 days to 1 month for larotrectinib (17/27, 63%), PBT (23/30, 77%) and tisagenlecleucel (19/30, 63%), as demonstrated in Table 4.

**Table 4:** Time, travel distance, and mode of transportation for patients and families to access blinatumomab, larotrectinib, proton therapy, and tisagenlecleucel, as reported by providers.

Time to Access	Blinatumomab (*N*, %)	Larotrectinib (*N*, %)	Proton Therapy (*N*, %)	Tisagenlecleucel (*N*, %)
*n*	35	27	30	30
1–5 days	17 (48.6%)	3 (11.1%)	2 (6.7%)	6 (20.0%)
5–14 days	15 (42.9%)	9 (33.3%)	11 (36.7%)	12 (40.0%)
14 days-1 month	2 (5.7%)	8 (29.6%)	12 (40.0%)	7 (23.3%)
>1 month	0 (0.0%)	1 (3.7%)	1 (3.3%)	0 (0.0%)

Distance	Blinatumomab (*N*, %)	Larotrectinib (*N*, %)	Proton Therapy (*N*, %)	Tisagenlecleucel (*N*, %)
*n*	N/A	N/A	30	30
<100 km	N/A	N/A	0 (0.0%)	13 (43.3%)
101–500 km	N/A	N/A	5 (16.7%)	6 (20.0%)
501–1,000 km	N/A	N/A	6 (20.0%)	4 (13.3%)
1,000 km-5,000 km	N/A	N/A	10 (33.3%)	2 (6.7%)
>5,000 km	N/A	N/A	5 (16.7%)	0 (0.0%)
Unsure	N/A	N/A	3 (10.0%)	1 (3.3%)

Mode of Transportation	Blinatumomab (*N*, %)	Larotrectinib (*N*, %)	Proton Therapy (*N*, %)	Tisagenlecleucel (*N*, %)
*n*	N/A	N/A	30	30
Air	N/A	N/A	20 (66.7%)	8 (26.7%)
Car	N/A	N/A	13 (43.3%)	20 (66.7%)
Bus	N/A	N/A	1 (3.3%)	1 (3.3%)
Train	N/A	N/A	0 (0.0%)	4 (13.3%)
Other	N/A	N/A	0 (0.0%)	2 (6.7%)
Unsure	N/A	N/A	2 (6.7%)	1 (3.3%)

Processes for accessing PBT and tisagenlecleucel were clearly defined for 28/30 (93.3%) and 27/30 (90%) of respondents respectively. 24/30 (80%) of respondents had a pediatric-specific CT centre that provided tisagenlecleucel within their province, with respondents from Nova Scotia and Saskatchewan highlighting the absence of such a centre in their province. Respondents also reported significant distances between their centre and centres providing both PBT and CT, with the most common mode of transportation being air for PBT (20/30, 66.7%), and car for CT (20/30, 66.7%), as described in Table 4.

### Funding sources

**Figure 1:**
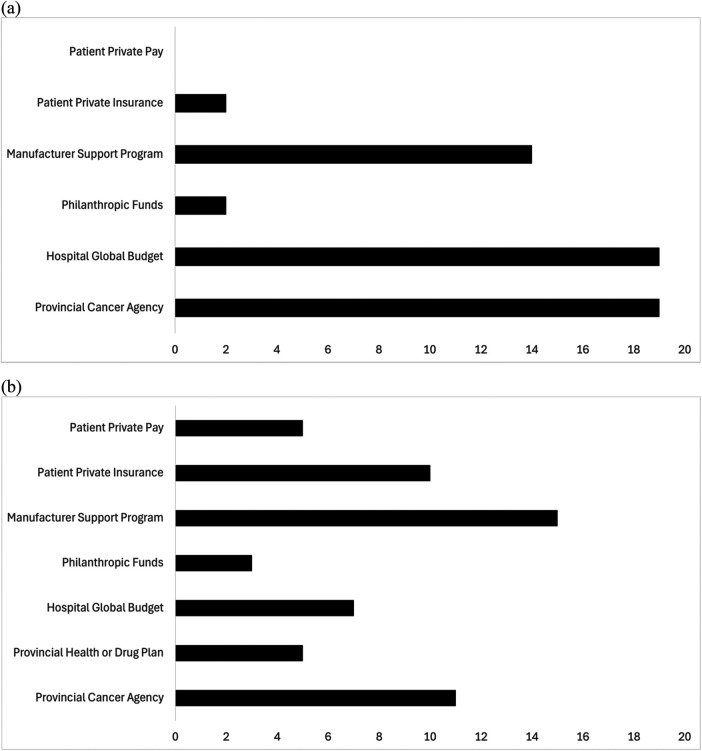

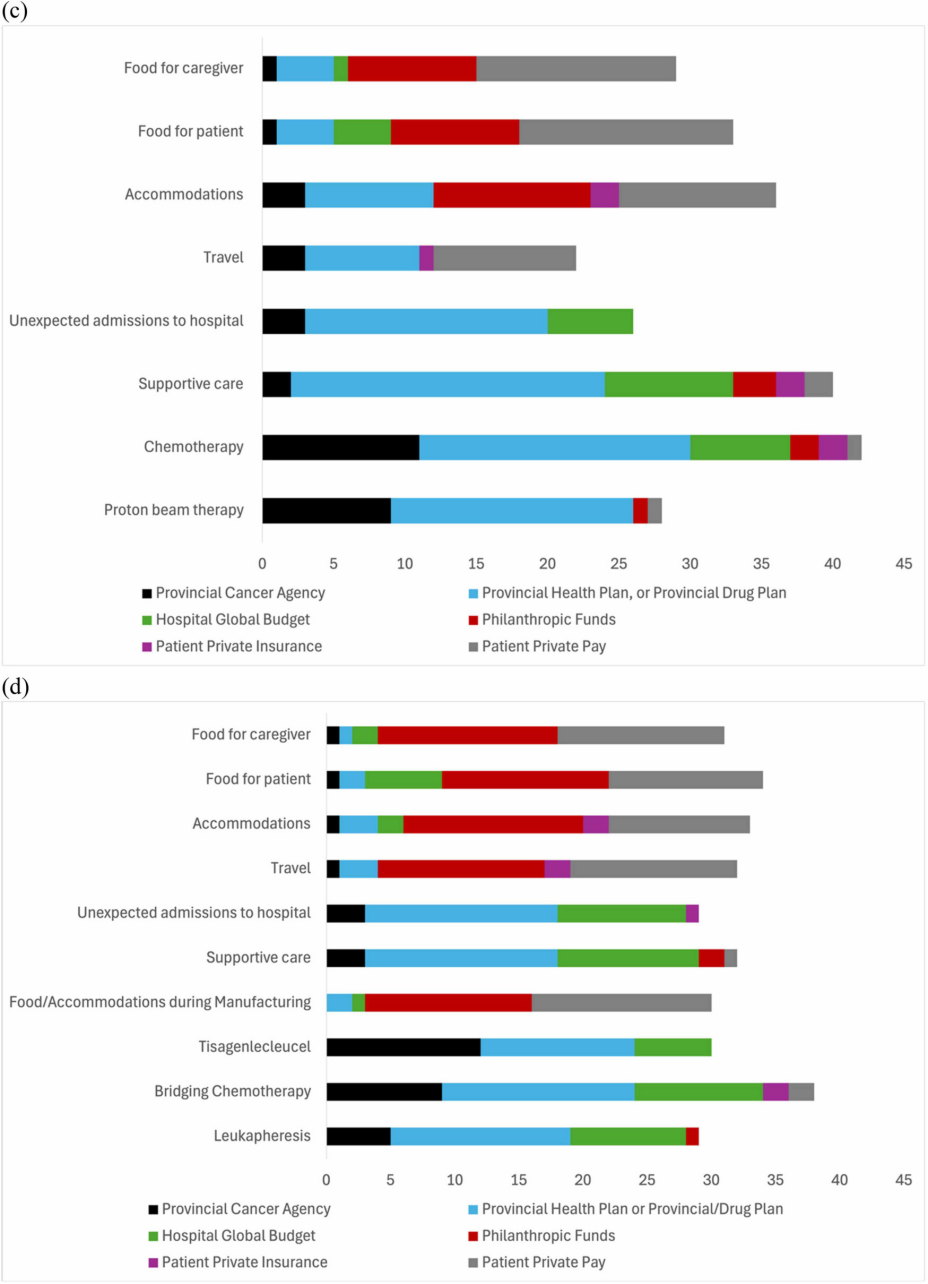
Funding sources identified by respondents for blinatumomab **(a)**, larotrectinib **(b)**, proton beam therapy and associated costs **(c)**, and tisagenlecleucel and associated costs **(d)**. The *x*-axis represents number of respondents. Totals may exceed the number of respondents because respondents could select all that apply.

Funding sources varied significantly based on the specific therapy and are summarized in Figure 1 and Supplementary Table 1. Primary sources of funding for blinatumomab and larotrectinib included provincial cancer agencies, hospital global budgets, and manufacturer support programs (Figures 1a,b). Patient private insurance was reported as a source of funding for larotrectinib for 10/27 (37%) of respondents (Figure 1b). Funding for PBT and tisagenlecleucel were primarily from provincial cancer agencies and provincial health plans, with hospital global budget being a source of funding tisagenlecleucel for 20% of respondents. 8/27 (26.7%) and 9/27 (30%) of respondents reported that the provincial health plan provided some funding for travel and accommodations respectively; however patient private pay and philanthropic funds were the most frequently selected source of funding for travel and accommodations (Figures 1c,d). Food for patient (15/30, 50%) and caregivers (14/30, 46.7%) while receiving PBT were identified as funded by patient private pay (Figure 1c). Additional costs of loss of income due to prolonged time away from work were also identified by respondents. Philanthropic funds were the most common source of funding for travel, accommodations, and food for patients and caregivers when accessing tisagenlecleucel (Figure 1d).

### Barriers to access

**Figure 2:**
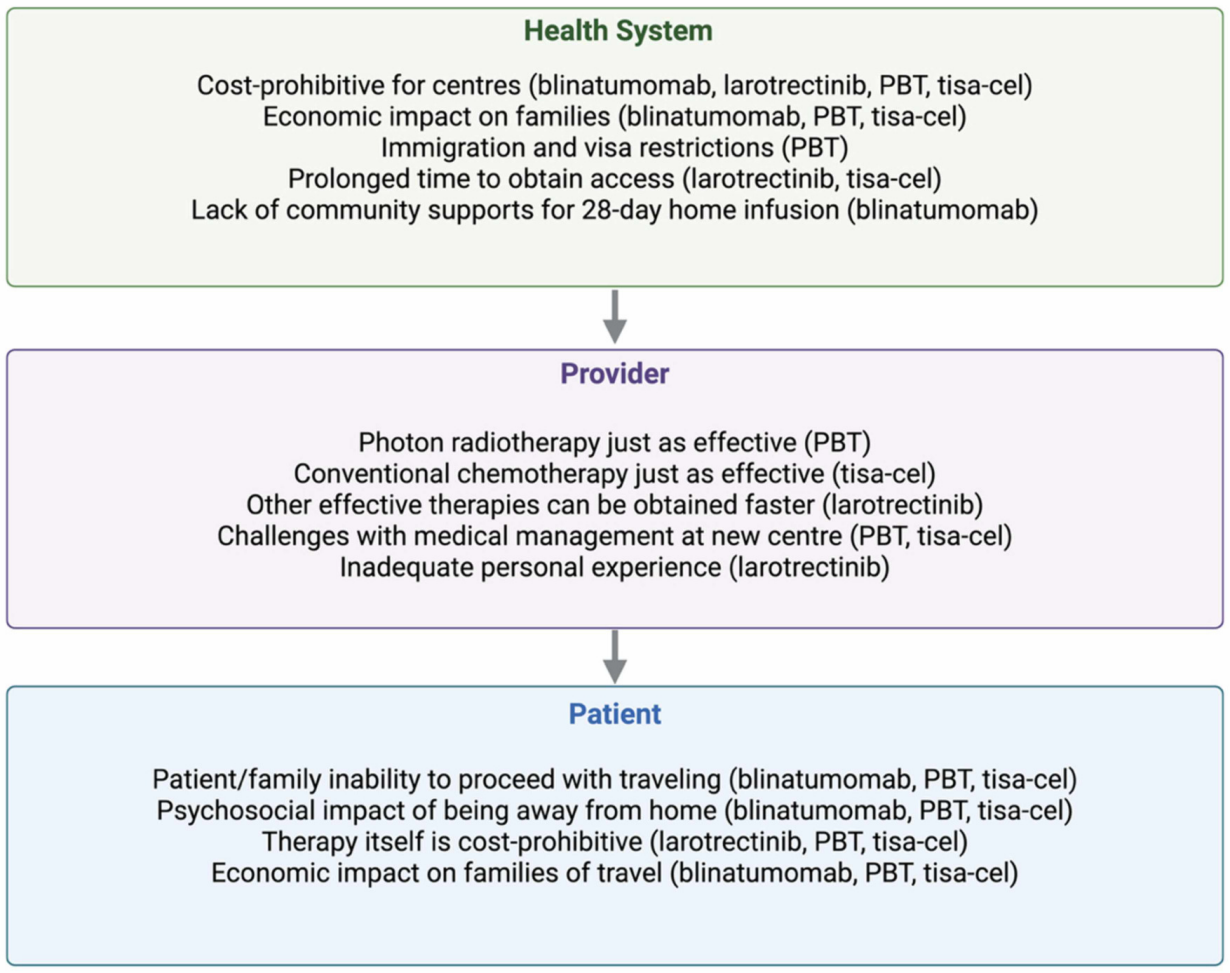
Conceptual representation of key barriers identified by respondents. Complete list of barriers and relative frequencies are included in Supplementary Table 2. Created in BioRender. PBT, proton beam therapy; tisa-cel, tisagenlecleucel.

Barriers to accessing each therapy are summarized in Figure 2 and detailed in Supplementary Table 2. Significant cost to patient (10/27, 37%) was the most frequently identified barrier in accessing larotrectinib, while patient/family inability to proceed with traveling was the most frequent barrier identified in accessing blinatumomab (7/35, 20%), PBT (19/30, 63%) and tisagenlecleucel (11/30, 36.7%). Immigration and visa restrictions (17/30, 57%), economic impact of travel (16/30, 53.3%), and psychosocial impact of travel (14/30, 46.7%), including due to patient complexity (16/30, 53.3%), were commonly highlighted barriers. Multiple participants reported no perceived barriers for each of the four therapies, with 14/35 (40%) reporting no perceived barriers when accessing blinatumomab. Prolonged time to obtaining therapy was more frequently identified with regards to PBT (10/30, 33%) and tisagenlecleucel (8/30, 27%), as compared to blinatumomab (5/35, 15%) and larotrectinib (3/27, 11%). Clinical trials were highlighted by two respondents as a successful avenue for accessing TRK inhibitors.

## Discussion

Our study highlights several key findings related to access to select high-cost cancer therapies for children in Canada, as reported by front-line oncology providers. While these therapies were reported to be accessible most of the time, there was marked variability across provinces, especially for PBT. Less variance was noted with tisagenlecleucel, with providers across provinces reporting access 70% of the time or greater. Our findings underscore that financial constraints and psychosocial impacts of traveling for therapy continue to impede equitable access in Canada. The selected evidence-informed therapies in this study are not uniformly funded by provincial and territorial bodies, resulting in variable degrees of expenses for families across jurisdictions.

Access concerns to blinatumomab have been identified globally, with one European survey reporting that only 40% of countries always had access to blinatumomab, highlighting the need for global strategies to ensure equitable access (20). While blinatumomab was felt to be accessible for low-risk relapse of B-ALL 89% of the time in this study, there exists significant variability across provinces, highlighting inequities despite availability. Additinally, key challenges with accessing blinatumomab became apparent from this study. KMT2A-rearranged infant B-ALL is a clear evidence-informed indication for blinatumomab (21), yet only 68.6% of respondents reported being able to access blinatumomab for this indication. This may demonstrate a gap in knowledge and institutional practice, or systemic challenges with accessing blinatumomab. This survey was designed prior to the results of the Children's Oncology Group's AALL1731 study results being published (22), and thus standard-risk B-ALL was not included as a possible indication in the survey. Improved overall survival in children with standard-risk B-ALL who receive blinatumomab has resulted and will continue to result in increased use of blinatumomab in upfront therapy (22), requiring clear avenues for accessing blinatumomab and supports for administering it. The CDA also provided a recommendation in 2025 for blinatumomab to be reimbursed for the treatment of patients with Ph-negative, CD19-positive B-ALL in the consolidation phase of multiphase chemotherapy (23). Barriers identified in this study, including lack of community supports for the 28-day infusion, and patient/family inability to travel to receive it must be addressed as blinatumomab becomes a cornerstone of therapy for children with B-ALL. These feasibility and infrastructure challenges were also highlighted as a criteria for reimbursement in the CDA recommendation for blinatumomab in first-line treatment (23). Funding sources for blinatumomab were primarily provincial cancer agencies, hospital global budgets, and manufacturer support programs. Although these funding sources seemingly eliminate costs for patients, the dependence on hospital global budget can lead to geographical inequities, and the growing use of blinatumomab highlights the need for more sustainable funding solutions, such as funding from the provincial health or drug plan.

Funding sources for larotrectinib were primarily provincial cancer agency, patient private insurance, and manufacturer support programs. Despite a clear recommendation for reimbursement from the CDA for larotrectinib, provincial health plans were not identified as a significant source of funding in this study, exposing a critical shortcoming in the healthcare system's responsibility to provide timely access to evidence-informed treatments (24). Manufacturer support programs have become a common method for accessing novel therapies in Canada, with compassionate access accounting for over 50% of all innovative drug requests in a recent retrospective study (25). The substantial potential cost to patients was identified as a barrier to accessing larotrectinib for those with TRK-positive tumors, underscoring the need for sustainable and equitable funding solutions. In addition, prolonged time to obtain larotrectinib as compared to other therapies that can be obtained faster was also a barrier. Thirty-eight percent of respondents noted that it took over 14 days to access larotrectinib, which can have significant impact on patient outcomes and highlights the importance of timely access. Delays in obtaining necessary and evidence-based therapies may not only impact patient outcomes (26), but may also deter oncologists from exploring accessing them, or these delays may result in urgent need to start a potentially less effective and more toxic therapy. Streamlining drug access processes, with sustainable sources of government funding would improve equitable and timely access to novel drugs like larotrectinib for children with cancer.

PBT was perceived as the least accessible therapy in this study, with only 59.2% of providers reporting they could access PBT for an unresectable head and neck sarcoma. Several factors may play into this, as identified in this study. Patients receiving PBT may incur several costs, which are inconsistently covered by provincial health care bodies. Respondents reported that proton beam therapy, chemotherapy, supportive care, and unexpected admissions were primarily funded by their provincial cancer agency, or provincial health or drug plan. In contrast, travel, accommodations, food for the patient and caregivers were identified as being funded by patient private pay or philanthropic funds. Additionally, 66% of respondents reported that air travel was the most common mode of transportation for PBT, for which costs may be enormous. Time away from work was highlighted as an additional potential cost to the family, which would affect patients and families from lower socioeconomic backgrounds more significantly. The economic burden of being in the US for several weeks to months is a massive barrier to access and disproportionately affects those from marginalized populations. These additional costs may deter Canadian providers and patients from considering PBT in the US, as they may feel that travel to the US, time away from work for caregivers, and the financial stress of being in the US may ultimately cause significant harm without much perceived benefit. This trend has been demonstrated in the US, with children from households with higher incomes and with private insurance being more likely to receive PBT in a retrospective study; it is not known, however, whether this trend due to referral patterns or different abilities to travel for PBT (3). Additionally, immigration/visa restrictions were noted to be a concern for a majority of respondents, highlighting the need for a domestic PBT facility.

There remains ongoing evidence suggesting comparable oncologic outcomes between photon and PBT (27, 28), and easier access to photon therapy within Canada, as highlighted by respondents in this study. These factors may ultimately result in the rejection of PBT as the best therapeutic option for patients who would clearly benefit from the decreased long-term toxicity offered by PBT. Although the intention of healthcare providers in these situations is to minimize harm, this ultimately results in vulnerable Canadian children being exposed to increased long-term toxicity and lower quality of life, further creating disparities.

Tisagenlecleucel and associated therapies (leukapheresis, bridging chemotherapy, supportive care, unexpected admissions) were reported to be funded primarily by provincial health and drug plans, provincial cancer agencies and hospital global budget. Similar to PBT, travel (predominantly by car), accommodations, and food for patient and caregivers were reported to be funded by philanthropic funds and patient private pay. Notably, providers in Nova Scotia and Saskatchewan reported a lack of a CT centre in the province, necessitating inter-provincial travel and therefore significant financial burden for families. For Nova Scotia, this is salient given the large catchment area that includes additional provinces, including New Brunswick and Prince Edward Island. Although travel within Canada does not present the immigration and visa challenges that accessing PBT abroad does, the psychosocial impact of being away from home remains significant. For both PBT and CT, families may fragment to continue a sustainable source of income, ultimately deepening inequities for vulnerable families. These families may not only lack access to effective therapy with decreased toxicity, but also face decreased quality of life due to family separation and resulting decreased family functioning (29, 30). Additionally, oncology providers in this study identified that patients may also have comorbidities beyond their oncologic diagnosis, making travelling to another city with unfamiliar medical teams particularly challenging.

Although prolonged time to accessing these therapies was highlighted as a barrier, complex processes to access these therapies were not a frequent barrier. This is consistent with over 90% of providers denoting a defined process for accessing PBT and tisagenlecleucel. In contrast, the economic and psychosocial impact of travel for these therapies are major barriers, especially for PBT, with the addition of visa and immigration restrictions. This contrast highlights that these provider perspectives on the process do not capture the entirety of challenges faced by patients and families.

While developing CT and PBT centres at all Canadian pediatric cancer centres in Canada will not likely be feasible nor economically viable, developing frameworks and systems that support families in accessing these therapies across Canada's significant geographic expanse is necessary. It has not been clearly demonstrated whether travel distance and rurality truly impact overall survival for children with cancer, but the increased financial and emotional strain of travel is well-documented (31). Our study highlights the need for sustainable sources of government funding for these therapies as well as significant psychosocial support for families as travel for these therapies is common within Canada. These economic and psychosocial barriers may contribute to oncology providers not offering evidence-informed and indicated therapy, ultimately leading to inequitable care. These trends have been seen in the US, with providers choosing not to offer PBT to patients of lower socioeconomic status, despite having the largest number of PBT centres (3, 32). This concerning finding underscores the importance of developing sustainable financial and psychosocial supports even when a Canadian proton beam centre is developed, as the development of a Canadian centre will not on its own alleviate the significant financial and emotional toxicity that patients and families experience when traveling for therapy. The US experience also shows that simply increasing the number of specialized centres does not guarantee greater equity. Establishing a centralized PBT Service in Canada, similar to England's approach, may be a strategy to streamline referrals and improve equitable PBT access for all age groups and diagnoses (33).

Our study has notable limitations. The true response rate is unclear due the unknown provider population reached by various distribution methods utilized. Most respondents resided in Ontario and Quebec, limiting jurisdictional comparisons. Social desirability bias may have influenced survey answers (34). Only available responses were analyzed, without imputation for missing data, resulting in potential nonresponse bias. Our survey only looked at specific provider perspectives and not objective data regarding access to these therapies. The reliance on perceived access in this study, as opposed to objective access metrics, may result in increased impact of recall bias and subjective interpretation, limiting the validity of these findings. As such, future research may include linking provider-reported access with administrative funding and treatment data to further clarify how policy decisions translate into real-world treatment delivery for children with cancer. These perspectives were obtained in reference to hypothetical vignettes and not actual clinical cases, which may have resulted in providers overestimating or underestimating access, and facilitators and barriers to access. Provider knowledge gaps were also noted in this study, and may have contributed to decreased access; as a result, these knowledge gaps may be a significant barrier to access, rather than system-level barriers. Access through clinical trials was also not explored in this study and may have had an impact on responses. Most importantly, this study did not examine the experiences of people with lived experience, or other key healthcare workers involved in drug and therapy access. The absence of patient perspectives and socioeconomic stratification of access is a critical gap and requires attention in future studies. Given that reluctance to travel for therapy is a common barrier identified in this study, future research should explore this aspect to better support patient-centred care.

Overall, our study highlights uneven access to key high-cost evidence-informed pediatric cancer therapies in Canada and identified notable barriers, including patient and family inability to travel, and the economic and psychosocial impact of travel. Such variations in access to these therapies challenge the notion of truly “universal” healthcare for children with cancer in Canada. Funding sources alleviated costs of direct therapies and supportive care, but costs of travel, accommodations, and food are not consistently supported, not only widening economic disparities in populations, but potentially impacting selection of therapies for patients, with possible implications for overall oncologic outcome and exposure to long-term toxicity. These provider-reported experiences from Canada offer a case study for other publicly funded health systems navigating this integration of high-cost, evidence-informed therapies into universal care models. Future research should investigate objective data demonstrating access to these therapies, patient perspectives on accessing these therapies, and implementation of system-level policy changes. Targeted policy changes, including travel supports, centralized referral models, and adoption of CDA reimbursement recommendations, are needed to address these financial and psychosocial burdens for patients and families. These policy implications will be especially important during the ongoing development of a Canadian proton beam centre, to ensure that access to these high-cost therapies is truly equitable for Canadian children with cancer.

## Previous publications

This work was previously published as a meeting abstract (online abstract publication) for the 2025 American Society of Clinical Oncology (ASCO) Annual Meeting on May 28, 2025, in the Journal of Clinical Oncology, Volume 42, Number 16 supplement. https://doi.org/10.1200/JCO.2025.43.16_suppl.e13500.

## Data Availability

The raw data supporting the conclusions of this article will be made available by the authors, without undue reservation.

## Supplementary material

Supplementary File S1 The survey instrument.

Supplementary Table S1 Funding sources for blinatumomab, larotrectinib, proton therapy, cellular therapy and associated costs.

Supplementary Table S2 Barriers to accessing each innovative therapy, as reported by oncology providers.
