# Supplementary material for: Understanding access to novel high-cost cancer therapies across Canada: a national survey of pediatric oncology providers
Source: Front Pediatr. 2026 May 20;14:1793250. doi: 10.3389/fped.2026.1793250 (PMC13229627; doi:10.3389/fped.2026.1793250)
Supplement: Supplementary File S1 — The survey instrument. [file Supplementaryfile1.docx]

# Access to Novel Cancer Therapies for Children - Survey

## Demographics

1. What is your primary role?
2. Pediatric medical oncologist
3. Radiation oncologist
4. Adult medical oncologist
5. Oncology pharmacist
6. Surgical oncologist
7. Hematopoietic stem cell transplant/cellular therapy physician
8. Nurse Practitioner/Advanced Practice Nurse
9. Other: _____________

A --> Scenario 1-4

B --> Scenario 3

C --> Scenario 1-4

D --> Scenario 1-2

E --> Scenario 1-4

F --> Scenario 4

G --> Scenario 1-4

H. Other --> Scenario 1-4 (with prompt to only fill out what is relevant to them)

1. In what province/territory do you primarily work?

- Yukon
- Northwest Territories
- Nunavut
- British Columbia
- Alberta
- Saskatchewan
- Manitoba
- Ontario
- Quebec
- Nova Scotia
- New Brunswick
- Prince Edward Island
- Newfoundland and Labrador

1. Which of the following centres do you primarily practice at?

- British Columbia Children’s Hospital
- Stollery Children’s Hospital
- Alberta Children’s Hospital
- Saskatoon Cancer Centre
- CancerCare Manitoba
- Children’s Hospital, London Health Sciences Centre
- McMaster Children’s Hospital
- Hospital for Sick Children
- Kingston General Hospital
- Children’s Hospital of Eastern Ontario
- CHU de Sherbrooke
- CHU Sainte-Justine
- Montreal Children’s Hospital
- IWK Health Centre
- Janeway Children's Health & Rehabilitation Centre
- Other: ______________
- None of the above

1. Approximately what percentage of the patients that you routinely care for are considered pediatric patients(Ie. under 18 years of age)?

- 80-100%
- 60-79%
- 40-59%
- 20-39%
- Less than 20%

1. How many years have you practiced in pediatric oncology, post-completion of your training?

- > 20 years
- 16-20 years
- 11-15 years
- 6-10 years
- Up to 5 years
- Currently in training

1. What is the population of the catchment area of the center you practice at?

- < 100000
- 100000 - 500000
- 500000 - 1 million
- 1 million – 3 million
- > 3 million
- Unsure: ______________

1. On average, how many children and youth at your centre are diagnosed with cancer every year?

- 0-25
- 25-50
- 50-100
- >100
- Unsure: ______________

1. What gender do you identify with?

- Male
- Female
- Non-binary
- Other: __________________
- Prefer not to answer

## Scenario 1

An 8-year old boy is being followed in your practice, who initially received therapy for standard-risk B-ALL at the age of 2. He presents now with bruising and is found to have pancytopenia and peripheral blasts. Bone marrow aspirate and diagnostic lumbar puncture confirms late combined bone marrow and central nervous system relapse of B-ALL (CNS 2). There is no evidence of testicular relapse. He receives re-induction with vincristine, mitoxantrone, dexamethasone, and Pegaspargase. At the end of induction, his minimal residual disease (MRD) is negative. Based on the low-risk arm of the recent Children’s Oncology Group (COG) trial AALL1331, you decide there is good evidence to support using three cycles of blinatumomab with conventional chemotherapy. The following questions pertain to the above scenario.

1. Is this scenario applicable to your clinical scope of practice?
   - Yes
   - No (move to next vignette)

**General**

1. Has your centre you used blinatumomab, for this specific indication (low-risk first bone marrow relapse of B-ALL)?
   - Yes
   - No, but have used blinatumomab for other indications
   - No, I have never used blinatumomab
2. For the child in the vignette, would you be able to offer blinatumomab at your centre, without cost to the family?

- Yes
- No
- Unsure: ______________

1. If not, could this patient travel to another centre for funded blinatumomab?

- Yes
- No
- Unsure: ______________

1. Approximately what percentage of patients with low-risk first relapse of B-ALL, who are MRD negative at the end of induction, would receive 3 cycles of blinatumomab as standard of care at your centre?
   - VAS 0-100%
2. Which of the following indications, if any, would you be able to access blinatumomab for (outside of a clinical trial)? (select all that apply)

- First relapse of B-ALL, MRD negative at End of Induction
- First relapse of B-ALL, MRD positive at End of Induction
- Infant KMT2A-rearranged B-ALL
- Bridging of front-line ALL therapy due to toxicity
- Salvage therapy for refractory B-ALL
- Other: ________________
- None of the above

**Obtaining Access**

1. Do you know what steps are required to obtain access to blinatumomab for the patient in the vignette at your centre?

- Yes
- No

1. Who is involved in obtaining access to blinatumomab at your centre? (select all that apply)

- Primary oncologist
- Pharmacist
- Social Worker
- Nurse Case Manager
- Drug Access Coordinator
- None, this is a pre-agreed upon indication on formulary
- Other: ___________

**Time to Access**

1. What is the typical time it would take to obtain access to blinatumomab from when the decision is made to administer it? Access in this scenario is defined as having the medication in hospital and ready for administration.
   - 1-5 days
   - 5-14 days
   - 14 days to 1 month
   - > 1 month
   - Other: ____________

**Cost of Therapy**

1. How would blinatumomab be funded **for this particular indication** at your centre? (select all that apply)
   - Provincial Cancer Agency
   - Hospital Global Budget
   - Philanthropic Funds
   - Manufacturer Support Program
   - Patient Private Insurance
   - Patient Private Pay
   - Other: _______________

**Barriers**

1. Which of the following barriers would stop you from accessing blinatumomab for the patient in the vignette? (select all that apply)

- No perceived barriers [all other options disappear]
- Prolonged time to obtain blinatumomab
- Cost prohibitive for treating centre
- Cost prohibitive for patient
- Inadequate equipment (i.e. CADD pump)
- Process for obtaining blinatumomab funding is too complex
- Other effective therapies can be obtained faster
- Inadequate personal experience with blinatumomab
- Inadequate process for administering blinatumomab
- Lack of community supports for 28-day home infusion
- Patient/family unwillingness to proceed with traveling for blinatumomab
- Patient complexity makes travel and medical management at new centre challenging
- Psychosocial impact on families of being away from home for several weeks
- Economic impact on families of being away from home for several weeks (e.g. away from job)
- Differences in medical management between proton beam facility and your centre
- Other: _______________________

## Scenario 2

A 13-year-old female presents with right thigh pain and swelling and is noted to have a thigh mass. Magnetic resonance imaging (MRI) demonstrates a 6cm x5cm mass in the right thigh. A biopsy is completed and is consistent with a spindle cell sarcoma. Staging investigations demonstrate localized disease with no metastases. Further testing reveals a TRK fusion in the primary thigh mass. You decide that proceeding with treatment with Larotrectinib monotherapy is the best course of management for this patient. The following questions pertain to the above scenario.

1. Is this scenario applicable to your clinical scope of practice?
   - Yes
   - No (move to next vignette)

**General**

1. Have you used larotrectinib at your centre, for this specific indication (TRK fusion positive sarcoma)?
   - Yes
   - No, but I have used larotrectinib for other indications
   - No, I have never used larotrectinib
2. For the child in this vignette, would you be able to offer larotrectinib at your centre, at minimal cost to the family?

- Yes
- No
- Unsure: ______________

1. If not, and the family were not able to afford larotrectinib out-of-pocket, would this patient receive conventional chemotherapy?
   - Yes
   - No
   - Unsure: ______________
2. Approximately what percentage of patients with a malignancy with a TRK fusion would receive Larotrectinib at minimal cost to the family at your centre?
   - VAS 0-100%
3. Which of the following diagnoses would you be able to access larotrectinib for (select all that apply)?
   - Infantile fibrosarcoma
   - Other TRK-fusion positive metastatic sarcomas
   - TRK-fusion positive CNS tumours
   - CNS tumours without a documented TRK-fusion
   - Papillary thyroid cancer
   - Other: _____________________
   - None of the above
4. In which of the following situations, if any, would you be able to access larotrectinib for NTRK fusion positive tumours? (select all that apply)
   - Metastatic disease
   - Localized tumour
   - Primary resection or radiation is not possible
   - First-line monotherapy
   - Monotherapy for NTRK fusion positive tumours that have failed to have a response with all standard of care therapies
   - Adjuvant use of larotrectinib with conventional chemotherapy as first-line therapy for NTRK fusion positive tumours
   - Adjuvant use of larotrectinib with conventional chemotherapy after failure of all standard of care therapies
   - Other: _____________________
   - None of the above

**Obtaining Access**

1. Do you know how to obtain access to larotrectinib for the patient at minimal cost in the vignette at your centre?

- Yes
- No

1. Who is involved in obtaining access to larotrectinib at your centre? (select all that apply)

- Primary oncologist
- Pharmacist
- Social Worker
- Nurse Case Manager
- Drug Access Coordinator
- Pharma Patient Assistance Program
- Other: ___________

1. Are there differences between obtaining access to therapies that are administered in an outpatient versus an inpatient setting?

- Yes
- No
- Unsure: ______________

1. If yes or unsure, please explain: ______________________________

**Time to Access**

1. What is the typical time it would take to obtain funded access to larotrectinib from when the decision is made to administer it? Access in this scenario is defined as having the medication ready for administration.
   - 1-5 days
   - 5-14 days
   - 14 days to 1 month
   - > 1 month
   - Other: _________________________

**Cost of Therapy**

1. Which of the following, if any, would provide support for funding of larotrectinib for the patient in the vignette? (select all that apply)
   - Provincial Cancer Agency
   - Hospital Global Budget
   - Philanthropic Funds
   - Manufacturer Support Program
   - Patient Private Insurance
   - Provincial Drug Plan (e.g. Ontario Drug Benefit Plan)
   - Patient Private Pay
   - Other: _______

**Barriers to Access**

1. Which of the following barriers would stop you from accessing larotrectinib for the patient in the vignette? (select all that apply)

- No perceived barriers [all other options disappear]
- Prolonged time to obtain larotrectinib
- Cost prohibitive for treating centre
- Cost prohibitive for patient
- Process for obtaining funding for larotrectinib is too complex
- Other effective therapies can be obtained faster
- Inadequate personal experience with larotrectinib
- Other: _______________________

## Scenario 3

A 2-year-old boy presents with left orbital swelling and initially receives a course of antibiotics for preseptal cellulitis. His swelling continues to progress and a CT scan of the orbits is completed demonstrating an orbital mass. The mass is biopsied, and he is diagnosed with Stage I, Group III embryonal rhabdomyosarcoma (translocation-negative) of the orbit. He begins to receive treatment with vincristine, dactinomycin, and cyclophosphamide. You decide to pursue radiotherapy given inability to resect the orbital rhabdomyosarcoma safely.

Based on the anatomy of the tumour in this patient, you determine that there is good evidence that proton beam therapy (proton therapy) is appropriate for this child to minimize potential long-term adverse effects of photon radiotherapy.

The following questions pertain to the scenario above.

1. Is this scenario applicable to your clinical scope of practice?
   - Yes
   - No (move to next vignette)

**General**

1. Do you have experience accessing **proton therapy** for this specific indication at your centre?
   - Yes
   - No, but have accessed proton therapy for another indication
   - No, have never accessed proton therapy
2. Would you pursue **proton therapy** for this patient in your practice?
   - Yes
   - No
   - Unsure: ______________
3. What percentage of patients at your centre with an unresectable sarcoma receive proton therapy?
   - VAS 0-100%
4. For which of the following indications in pediatric patients, if any, would you be able to access proton therapy? (select all that apply)
   - Head-and-neck solid tumours
   - CNS tumours
   - Ocular tumours
   - Patients with cancer predisposition syndromes (increased radiosensitivity)
   - Retroperitoneal tumours
   - Pelvic and genitourinary tumours
   - Other: _____________
   - None of the above

**Obtaining Access**

As there are currently no hospital-based proton beam facilities operating in Canada, you plan to send this child and his family out of the country to receive proton therapy.

1. Do you know the steps in the process to obtain funding to send a child for proton therapy?

- Yes
- No

1. Who is involved in obtaining applying to your provincial health agency or Ministry of Health for funding support towards proton therapy at your centre? (select all that apply)

- Pediatric oncologist
- Radiation oncologist
- Pharmacist
- Social Worker
- Nurse Case Manager
- Other: ___________________

1. Does your jurisdiction have a defined process for approval for funding for proton therapy?
   - Yes
   - No
2. If no, please describe the process for approval: _______________________________________
3. Does your centre have preferred proton beam facilities?
   - Yes
   - No
4. If yes, how many?

- 1
- 2-3
- 4-5
- >5
- Not sure

1. Could you estimate the distance from your centre to your closest preferred proton beam facility?

- < 100km
- 101-500km
- 501-1000km
- 1000km-5000km
- > 5000km
- Unsure: ______________

1. How do families travel to the proton beam facility you refer to most commonly?

- Air
- Car
- Bus
- Train
- Other: _________
- Unsure: ______________

1. In which city is your preferred proton beam facility located? _________________________

**Time to Access**

1. What is the typical time (calendar days) it would take to obtain approval for funding for proton therapy?
   - 1-5 days
   - 5-14 days
   - 14 days to 1 month
   - > 1 month
   - Other: __________________

**Cost of Therapy**

1. How would costs of the items below be funded for the patient in the vignette? For each item listed, please select all the sources of funding that would be available for the patient in the vignette. For example, if food is funded by both patient private pay and the hospital global budget, please select both sources of funds.

|  | Provincial Cancer Agency | Provincial Health Plan | Hospital Global Budget | Philanthropic Funds | Patient Private Insurance | Patient Private Pay | Unsure: ______________ | Other Source of Funding (please explain in comments) |
| --- | --- | --- | --- | --- | --- | --- | --- | --- |
| Proton beam therapy |  |  |  |  |  |  |  |  |
| Chemotherapy |  |  |  |  |  |  |  |  |
| Supportive care (e.g. transfusions, IV fluids, nutrition) |  |  |  |  |  |  |  |  |
| Unexpected admissions to hospital |  |  |  |  |  |  |  |  |
| Travel |  |  |  |  |  |  |  |  |
| Accommodations |  |  |  |  |  |  |  |  |
| Food for patient |  |  |  |  |  |  |  |  |
| Food for caregiver |  |  |  |  |  |  |  |  |
| Other potential cost to family (please explain in comments) |  |  |  |  |  |  |  |  |

**Barriers to Access**

1. Which of the following barriers could stop you from pursuing proton therapy for the patient in the vignette? (select all that apply)

- No perceived barriers [all other options disappear]
- Prolonged time to obtain approval for proton therapy
- Distance of proton beam facility from your centre
- Direct costs of proton therapy prohibitive for your centre
- Direct costs of proton therapy prohibitive for patient
- Immigration/visa restrictions for patient and/or family
- Process for obtaining approval for proton therapy is too complex or takes too much time
- Process for obtaining approval for proton therapy is unfunded (i.e. no reimbursement for physician paperwork)
- Photon radiotherapy easier to obtain
- Photon radiotherapy is just as effective
- Inadequate personal experience with proton therapy
- Patient/family unwillingness to proceed with traveling for proton therapy
- Patient complexity makes travel and medical management at new centre challenging
- Psychosocial impact on families of being away from home for several weeks
- Economic impact on families of being away from home for several weeks (e.g. away from job)
- Differences in medical management between proton beam facility and your centre
- Funding only available for proton beam therapy and chemotherapy but not for unexpected hospital admissions
- Other: _______________________

## Scenario 4

A 14 year-old patient with Trisomy 21 is being followed in your practice, who had precursor B-acute lymphoblastic leukemia (B-ALL) at the age of 10 years. She received therapy per your standard of care for high-risk B-ALL patients with Down’s Syndrome due to age on presentation. There was no significant toxicity during treatment, and she completed therapy 24 months ago. During a routine follow up she is found to have peripheral blasts. Investigations confirm a late combined bone marrow and central nervous system relapse of B-ALL.

You discuss options for further treatment with multiple colleagues and decide to explore using tisagenlecleucel (Kymriah) as curative therapy for this patient. There is no suitable clinical trial available for this patient both locally or nationally.

1. Is this scenario applicable to your clinical scope of practice?
   - Yes
   - No (move to next vignette)

**General**

1. Do you have experience accessing CAR-T Cell Therapy (tisagenlecleucel) for this specific indication at your centre?
   - Yes
   - No, but have accessed tisagenlecleucel for another indication
   - No, have never accessed tisagenlecleucel
2. For the child in this vignette, would your centre be able to offer CAR-T Cell Therapy (tisagenlecleucel) either in your centre, or at a partner facility in or out of province?

- Yes
- No
- Unsure: ______________

1. What percentage of patients at your centre with Down’s syndrome and first relapse of B-ALL would receive tisagenlecleucel?
   - VAS 0-100%
2. For which of the following indications in pediatric patients, if any, would you be able to access tisagenlecleucel? (select all that apply)
   - Second or greater relapse of B-ALL following hematopoietic stem cell transplant
   - First relapse of B-ALL in patients with Down’s Syndrome
   - CNS positive relapse of B-ALL in very young patients (< 3 years)
   - Primary refractory B-ALL
   - B-ALL with positive MRD at the end of consolidation
   - Other: ____________________
   - None of the above

**Obtaining Access**

1. Do you know the steps in the process to obtain funding to send a child for cellular therapy?

- Yes
- No

1. Does the above process differ for a child with an approved Health Canada indication (second relapse of B-ALL) compared to off-label use of tisagenlecleucel?
   - Yes
   - No
   - Unsure: ______________
2. Who is involved in obtaining access to cellular therapy?

- Primary oncologist
- Pharmacist
- Social Worker
- Nurse Case Manager
- Transplant Physician at Cellular Therapy Centre
- Other: _______

1. Does your jurisdiction have a defined process for approval for funding for cellular therapy?
   - Yes
   - No
2. If no, please describe the process for approval: _______________________________________
3. Is there a pediatric-specific cellular therapy centre that provides tisagenlecleucel in your province?

- Yes
- No
- Unsure: ______________

1. How far from your centre is the cellular therapy centre you most commonly refer to?

- < 100km
- 101-500km
- 501-1000km
- >1000km

1. How do families travel to the cellular therapy centre you most commonly refer to?

- Air
- Car
- Bus
- Train
- Other: _________
- Unsure: ______________

**Time to Access**

1. What is the typical time it would take to obtain approval for cellular therapy for the patient in the vignette?
   - 1-5 days
   - 5-14 days
   - 14 days to 1 month
   - > 1 month
   - Other: ________________

**Cost of Therapy**

1. How would costs of the items below be funded for the patient in the vignette? For each item listed, please select all the sources of funding that would be available for the patient in the vignette. For example, if food is funded by both patient private pay and the hospital global budget, please select both sources of funds.

|  | Provincial Cancer Agency | Provincial Health Plan | Hospital Global Budget | Philanthropic Funds | Patient Private Insurance | Patient Private Pay | Unsure: ______________ | Other Source of Funding (please explain in comments) | Not applicable |
| --- | --- | --- | --- | --- | --- | --- | --- | --- | --- |
| Leukapheresis |  |  |  |  |  |  |  |  |  |
| Bridging Chemotherapy |  |  |  |  |  |  |  |  |  |
| Car-T Cell Therapy |  |  |  |  |  |  |  |  |  |
| Food and Accommodations during Manufacturing Period |  |  |  |  |  |  |  |  |  |
| Supportive care (e.g. transfusions, IV fluids, nutrition) |  |  |  |  |  |  |  |  |  |
| Unexpected admissions to hospital |  |  |  |  |  |  |  |  |  |
| Travel |  |  |  |  |  |  |  |  |  |
| Accommodations |  |  |  |  |  |  |  |  |  |
| Food for patient |  |  |  |  |  |  |  |  |  |
| Food for caregiver |  |  |  |  |  |  |  |  |  |
| Other potential cost to family |  |  |  |  |  |  |  |  |  |

**Barriers to Access**

1. Which of the following barriers would stop you from pursuing cellular therapy for the patient in the vignette? (select all that apply)

- No perceived barriers [all other options disappear]
- Prolonged time to obtain access to cellular therapy
- No access to leukapheresis while approval for cellular therapy is pending
- Distance to cellular therapy centre
- Cost prohibitive for treating centre
- Cost prohibitive for patient
- Immigration/visa restrictions for patient and/or family
- Process for obtaining approval for CAR-T cell therapy is too complex or takes too much time
- Process for obtaining approval for cellular therapy is unfunded (i.e. no reimbursement for physician paperwork)
- Conventional chemotherapy can be just as effective
- Inadequate personal experience with cellular therapy
- Patient/family unwillingness to proceed with traveling for cellular therapy
- Patient complexity makes travel and medical management at new centre challenging
- Psychosocial impact on families of being away from home for several weeks
- Economic impact on families of being away from home for several weeks (e.g. away from job)
- Differences in medical management between cellular therapy centre and your centre
- Funding only available for cellular therapy and chemotherapy but not for unexpected hospital admissions
- Funding for intensive care unit admissions and anti-cytokine therapies
- Other: _______________________

## Other

1. Is there anything else you would like to share? (blank text)
2. If you would like to be contacted about further research in this area, please include your contact information here. Your contact information will not be linked to your answers.
