# Supplementary material for: Understanding access to novel high-cost cancer therapies across Canada: a national survey of pediatric oncology providers
Source: Front Pediatr. 2026 May 20;14:1793250. doi: 10.3389/fped.2026.1793250 (PMC13229627; doi:10.3389/fped.2026.1793250)
Supplement: Supplementary Table S1 — Funding sources for blinatumomab, larotrectinib, proton therapy, cellular therapy and associated costs. [file Table1.docx]

Supplemental Table 1: Funding sources for blinatumomab, larotrectinib, proton therapy, cellular therapy and associated costs.

|  | Provincial Cancer Agency | Provincial Health Plan, or Provincial Drug Plan | Hospital Global Budget | Philanthropic Funds | Manufacturer Support Program | Patient Private Insurance | Patient Private Pay | Other |
| --- | --- | --- | --- | --- | --- | --- | --- | --- |
| *Blinatumomab, n=35* | | | | | | | | |
| Blinatumomab | 19 (54.3%) | N/A | 19 (54.3%) | 2 (5.7%) | 14 (40.0%) | 2 (5.7%) | 0 (0.0%) | 0 (0.0%) |
| *Larotrectinib, n=27* | | | | | | | | |
| Larotrectinib | 11 (40.7%) | 5 (18.5%) | 7 (25.9%) | 3 (11.1%) | 15 (55.6%) | 10 (37.0%) | 5 (18.5%) | 0 (0.0%) |
| *Proton therapy and additional potential costs, n=30* | | | | | | | | |
| Proton beam therapy | 9 (30.0%) | 17 (56.7%) | 0 (0.0%) | 1 (3.3%) | N/A | 0 (0.0%) | 1 (3.3%) | 0 (0.0%) |
| Chemotherapy | 11 (36.7%) | 19 (63.3%) | 7 (23.3%) | 2 (6.7%) | N/A | 2 (6.7%) | 1 (3.3%) | 0 (0.0%) |
| Supportive care (e.g. transfusions, intravenous fluids, nutrition) | 2 (6.7%) | 22 (73.3%) | 9 (30.0%) | 3 (10.0%) | N/A | 2 (6.7%) | 2 (6.7%) | 0 (0.0%) |
| Unexpected admissions to hospital | 3 (10.0%) | 17 (56.7%) | 6 (20.0%) | 0 (0.0%) | N/A | 0 (0.0%) | 0 (0.0%) | 0 (0.0%) |
| Travel | 3 (10.0%) | 8 (26.7%) | 0 (0.0%) | 9 (30.0%) | N/A | 1 (3.3%) | 10 (33.3%) | 3 (10.0%) |
| Accommodations | 3 (10.0%) | 9 (30.0%) | 0 (0.0%) | 11 (36.7%) | N/A | 2 (6.7%) | 11 (36.7%) | 4 (13.3%) |
| Food for patient | 1 (3.3%) | 4 (13.3%) | 4 (13.3%) | 9 (30.0%) | N/A | 0 (0.0%) | 15 (50.0%) | 3 (10.0%) |
| Food for caregiver | 1 (3.3%) | 4 (13.3%) | 1 (3.3%) | 9 (30.0%) | N/A | 0 (0.0%) | 14 (46.7%) | 4 (13.3%) |
| *Tisagenlecleucel and potential additional costs, n=30* | | | | | | | | |
| Leukapheresis | 5 (16.7%) | 14 (46.7%) | 9 (30.0%) | 1 (3.3%) | N/A | 0 (0.0%) | 0 (0.0%) | 0 (0.0%) |
| Bridging Chemotherapy | 9 (30.0%) | 15 (50.0%) | 10 (33.3%) | 0 (0.0%) | N/A | 2 (6.7%) | 2 (6.7%) | 0 (0.0%) |
| Tisgenlecleucel | 12 (40.0%) | 12 (40.0%) | 6 (20.0%) | 0 (0.0%) | N/A | 0 (0.0%) | 0 (0.0%) | 0 (0.0%) |
| Food and Accommodations during Manufacturing Period | 0 (0.0%) | 2 (6.7%) | 1 (3.3%) | 13 (43.3%) | N/A | 0 (0.0%) | 14 (46.7%) | 6 (20.0%) |
| Supportive care (e.g. transfusions, intravenous fluids, nutrition) | 3 (10.0%) | 15 (50.0%) | 11 (36.7%) | 2 (6.7%) | N/A | 0 (0.0%) | 1 (3.3%) | 0 (0.0%) |
| Unexpected admissions to hospital | 3 (10.0%) | 15 (50.0%) | 10 (33.3%) | 0 (0.0%) | N/A | 1 (3.3%) | 0 (0.0%) | 0 (0.0%) |
| Travel | 1 (3.3%) | 3 (10.0%) | 0 (0.0%) | 13 (43.3%) | N/A | 2 (6.7%) | 13 (43.3%) | 5 (16.7%) |
| Accommodations | 1 (3.3%) | 3 (10.0%) | 2 (6.7%) | 14 (46.7%) | N/A | 2 (6.7%) | 11 (36.7%) | 6 (20.0%) |
| Food for patient | 1 (3.3%) | 2 (6.7%) | 6 (20.0%) | 13 (43.3%) | N/A | 0 (0.0%) | 12 (40.0%) | 5 (16.7%) |
| Food for caregiver | 1 (3.3%) | 1 (3.3%) | 2 (6.7%) | 14 (46.7%) | N/A | 0 (0.0%) | 13 (43.3%) | 6 (20.0%) |
