# Supplementary material for: Understanding access to novel high-cost cancer therapies across Canada: a national survey of pediatric oncology providers
Source: Front Pediatr. 2026 May 20;14:1793250. doi: 10.3389/fped.2026.1793250 (PMC13229627; doi:10.3389/fped.2026.1793250)
Supplement: Supplementary Table S2 — Barriers to accessing each innovative therapy, as reported by oncology providers. [file Table2.docx]

Supplemental Table 2: Barriers to accessing each innovative therapy, as reported by oncology providers.

| ***Blinatumomab, n =35*** | ***N (%)*** |
| --- | --- |
| No perceived barriers | 14 (40.0%) |
| Prolonged time to obtain blinatumomab | 5 (14.3%) |
| Cost prohibitive for treating centre | 5 (14.3%) |
| Cost prohibitive for patient | 2 (5.7%) |
| Inadequate equipment (i.e. infusion pump) | 3 (8.6%) |
| Process for obtaining blinatumomab funding is too complex | 0 (0.0%) |
| Other effective therapies can be obtained faster | 3 (8.6%) |
| Inadequate personal experience with blinatumomab | 1 (2.9%) |
| Inadequate process for administering blinatumomab | 0 (0.0%) |
| Lack of community supports for 28-day home infusion | 5 (14.3%) |
| Patient/family inability to proceed with traveling for blinatumomab | 7 (20.0%) |
| Patient complexity makes travel and medical management at new centre challenging | 1 (2.9%) |
| Psychosocial impact on families of being away from home for several weeks | 6 (17.1%) |
| Economic impact on families of being away from home for several weeks (e.g. away from job) | 5 (14.3%) |
| Differences in medical management between centre providing blinatumomab and your centre | 0 (0.0%) |
|  |  |
| ***Larotrectinib, n=27*** | ***N (%)*** |
| No perceived barriers | 4 (14.8%) |
| Prolonged time to obtain larotrectinib | 3 (11.1%) |
| Cost prohibitive for treating centre | 5 (18.5%) |
| Cost prohibitive for patient | 10 (37.0%) |
| Process for obtaining funding for larotrectinib is too complex | 1 (3.7%) |
| Other effective therapies can be obtained faster | 6 (22.2%) |
| Inadequate personal experience with blinatumomab | 3 (11.1%) |
| Other (including private insurance denial, unsure) | 2 (7.4%) |
|  |  |
| ***Proton Beam Therapy, n = 30*** | ***N (%)*** |
| No perceived barriers | 1 (3.3%) |
| Prolonged time to obtain approval for proton therapy | 10 (33.3%) |
| Distance of proton beam facility from your centre | 7 (23.3%) |
| Direct costs of proton therapy prohibitive for your centre | 2 (6.7%) |
| Direct costs of proton therapy prohibitive for patient | 9 (30.0%) |
| Immigration/visa restrictions for patient and/or family | 17 (56.7%) |
| Process for obtaining approval for proton therapy is too complex or takes too much time | 3 (10.0%) |
| Process for obtaining approval for proton therapy is unfunded (i.e. no reimbursement for physician paperwork) | 0 (0.0%) |
| Photon radiotherapy easier to obtain | 11 (36.7%) |
| Photon radiotherapy is just as effective | 11 (36.7%) |
| Inadequate personal experience with proton therapy | 1 (3.3%) |
| Patient/family inability to proceed with traveling for proton therapy | 19 (63.3%) |
| Patient complexity makes travel and medical management at new centre challenging | 16 (53.3%) |
| Psychosocial impact on families of being away from home for several weeks | 14 (46.7%) |
| Economic impact on families of being away from home for several weeks (e.g. away from job) | 16 (53.3%) |
| Differences in medical management between proton beam facility and your centre | 4 (13.3%) |
| Funding only available for proton beam therapy and chemotherapy but not for unexpected hospital admissions | 3 (10.0%) |
|  |  |
| ***Tisagenlecleucel, n=30*** | ***N (%)*** |
| No perceived barriers | 5 (16.7%) |
| Prolonged time to obtain access to tisagenlecleucel | 8 (26.7%) |
| No access to leukapheresis while approval for tisagenlecleucel is pending | 3 (10.0%) |
| Distance to tisagenlecleucel centre | 2 (6.7%) |
| Cost prohibitive for treating centre | 3 (10.0%) |
| Cost prohibitive for patient | 3 (10.0%) |
| Immigration/visa restrictions for patient and/or family | 3 (10.0%) |
| Process for obtaining approval for tisagenlecleucel is too complex or takes too much time | 1 (3.3%) |
| Process for obtaining approval for tisagenlecleucel is unfunded (i.e. no reimbursement for physician paperwork) | 1 (3.3%) |
| Conventional chemotherapy can be just as effective | 3 (10.0%) |
| Inadequate personal experience with tisagenlecleucel | 0 (0.0%) |
| Patient/family inability to proceed with traveling for tisagenlecleucel | 11 (36.7%) |
| Patient complexity makes travel and medical management at new centre challenging | 4 (13.3%) |
| Psychosocial impact on families of being away from home for several weeks | 5 (16.7%) |
| Economic impact on families of being away from home for several weeks (e.g. away from job) | 5 (16.7%) |
| Differences in medical management between cellular therapy centre and your centre | 1 (3.3%) |
| Funding only available for tisagenlecleucel and chemotherapy but not for unexpected hospital admissions | 0 (0.0%) |
| Funding not available for intensive care unit admissions and anti-cytokine therapies | 1 (3.3%) |
